# Engagement and learning in an electronic spaced repetition curriculum companion for a paediatrics academic half-day curriculum

**DOI:** 10.1007/s40037-021-00680-x

**Published:** 2021-09-13

**Authors:** Jason R. McConnery, Ereny Bassilious, Quang N. Ngo

**Affiliations:** 1 Department of Paediatric Respiratory Medicine, The Hospital for Sick Children, Toronto ON, Canada; 2 Department of Paediatrics, McMaster University Medical Centre, Hamilton ON, Canada

**Keywords:** Medical education, Residency, Paediatrics, Spaced repetition, Learning

## Abstract

Postgraduate residencies utilize academic half-days to supplement clinical learning. Spaced repetition reinforces taught content to improve retention. We leveraged spaced repetition in a curriculum companion for a paediatric residency program’s half-day. One half-day lecture was chosen weekly for reinforcement (day 0). Participants received 3 key points on day 1 and a multiple-choice question (MCQ) on day 8. On day 29, they received two MCQs to test reinforced and unreinforced content from the same day 0. Thirty-one (79%) residents participated over 17 weeks, but only 14 (36%) completed more than half of the weekly quizzes. Of all quizzes, 37.4% were completed, with an average weekly engagement of 5.5 minutes. Helpfulness to learning was rated as 7.89/10 on a Likert-like scale. Reported barriers were missing related half-days and emails, or limited time. There was no significant difference in performance between reinforced (63.4%, [53.6–73.3]) and unreinforced (65.6%, [53.7–73.2]) questions. Spaced repetition is a proven strategy in learning science, but was not shown to improve performance. Operational barriers likely limited participation and underpowered our analysis, therefore future implementation must consider practical and individual barriers to facilitate success. Our results also illustrate that satisfaction alone is an inadequate marker of success.

**Supplementary Information:**

The online version of this article (10.1007/s40037-021-00680-x) contains supplementary material, which is available to authorized users.

## The story

Much of learning in a residency program occurs at the bedside, in rounds, and through other clinical teaching methods. This approach, however, relies heavily on chance to achieve learning objectives set by specialty education regulatory bodies. To supplement clinical learning and address gaps in clinical education, most programs employ didactic methods such as the academic half-day. Our program utilizes a typical, in-person didactic format of three lectures in three hours during which general or subspecialist paediatricians deliver lectures, with variable inclusion of slides, case examples, and audience-participation questions, to achieve the Royal College of Physicians and Surgeons of Canada training objectives for paediatrics. Unfortunately, important supports to learning in a didactic academic half-day, such as independent pre-reading and post-lecture review, can be challenging due to time constraints outside of protected hours. As a result, organized studying is often neglected until the pressures of board examination preparation rise, as reflected by high pass rates on board examinations compared with in-training exams [1, 2]. Recognition of this pattern has increased discussions about techniques to improve learner engagement and learning effectiveness, including leveraging advances in technology, such as Free-Open Access Medical Education (FOAMed) and study apps, to address this problem [3].

Spaced repetition is an evidence-based learning technique based on the spacing effect, first described by Ebbinghaus in 1885. It suggests that retention of learned content decays rapidly without frequent review [4]. Spaced repetition has been shown to improve retention and slow this natural rate of decay for recall of newly learned material. This strategy stands in contrast to a less efficient repetition interval: massed repetition, otherwise known as cramming [5]. Spaced repetition also takes advantage of the testing effect, which suggests active review of knowledge that challenges the learner’s retention is more effective than simply memorizing facts [6, 7]. In undergraduate medical education, spaced repetition increases topic-specific learning to improve test scores [8]. In postgraduate and continuing medical education (CME), spaced repetition improves acquisition and retention of discrete topics of medical knowledge [9, 10]. In CME, spaced repetition increases self-reported changes in clinical behaviour, suggesting efficacy in translating instruction to practice [11].

The purpose of this study was to leverage spaced repetition and testing as a curriculum companion to reinforce a paediatric residency program’s academic half-day curriculum. As a primary objective, we wanted to illustrate resident engagement in a novel educational intervention and query satisfaction with the study tool, with the secondary objective being to assess its effectiveness in improving learning.

To do so, all postgraduate year (PGY) 1, 2 and 3 residents undertaking paediatric postgraduate training at McMaster University were enrolled. Participation was weekly on an opt-out basis. Thirty-nine residents were eligible. Our study received exemption from the Research Ethics Board at McMaster University.

We delivered all the instruments via email and administered the curriculum companion through a Google Form, containing challenge questions related to half-day material, and immediate feedback on performance. On day 1, we reinforced core content from one academic half-day session (delivered on the previous day; day 0) using a key points summary slide provided by the lecturer and a prompted reflection on how content would change the resident’s practice. On day 8, we delivered a lecturer-generated question to challenge learner retention and understanding of the topic. Finally, on day 29, we delivered a new question challenging retention of the topic once more, along with another challenge question on a concept from an unreinforced academic half-day session from the same day 0. Day 29 questions were drawn from a large question bank developed by Canadian Paediatric Program Directors and used for in-training exams. We selected this as our question source for their markers of validity by Messick’s framework [12]; questions from this bank are developed by content experts, used bi-annually with normal distribution among trainees, subject to response process where problematic questions are revised or removed, and show improvement in performance by postgraduate level. Importantly, a pass on the exams these questions were drawn from is 70%. Specific questions were selected from this bank on the basis of applicability to the lecture content presented on day 0.

After submitting an answer to each question (day 8 or 29), the resident was immediately provided feedback on correct and incorrect responses and offered links to resources for optional further study. We repeated this cycle weekly for 17 iterations such that participants, at maximum, received one summary slide (day 1) and completed and received feedback on three questions per week (day 8 and day 29 of respective reinforced/unreinforced academic half-day sessions). This was intended to take no more than 5–10 minutes, although optional links (review articles, educational videos, etc.) to extend learning engagement were made available. We chose these intervals to take advantage of expanding retrieval whereby the interval between challenges increases with each subsequent challenge [13], similarity to scientifically proven spacing algorithms [5], and its ability to fit within our weekly academic half-day framework. Over the first 13 weeks of the study, there were 11 reinforced and unreinforced lectures (two half-days were non-didactic). The remaining four weeks allowed completion of the last four reinforcement cycles (day 29 questions following weeks 9–13).

Residents self-identified using an anonymized number. We collected time spent each week as a measure of engagement (reported start time; end time via the form submission time stamp). Performance on day 8 questions was considered formative and not analyzed. Performance on day 29 challenge questions was pooled for analysis. Finally, a post-study questionnaire was emailed seeking feedback and learner experience regarding the learning intervention, perceptions of learning, and barriers to use.

We analysed whole group and sub-group (by PGY) scores for day 29 questions on reinforced content compared with unreinforced content, using an unpaired two-tailed student’s t‑test. With an alpha of 0.05, a desired power of 0.80, and a standard deviation of 8.5% (average standard deviation on four most recent in-training MCQ exams), our study required a sample size of 16 to detect a difference of one standard deviation (8.5%) in exam scores. Statistical analysis was performed using Microsoft Excel. We analysed overall resident satisfaction with the curriculum companion based on objective engagement data and the post-study questionnaire. Likert-like scale responses were averaged, and qualitative responses examined for common themes.

## Surprising outcomes

Out of 39 participants, 31 (79.0%) tried the tool at least once; however, participation quickly dropped off. Just 22 (56.0%) participated on at least four occasions, and only 14 (36.0%) participated for more than half (≥ 9) of the weekly quizzes. The highest participation overall was amongst PGY3 residents (83.0% trying at least once); however more PGY1 residents consistently used the tool, with 46.0% completing over half of the weekly quizzes. Disappointingly, only two residents completed all 17 quizzes.

Out of 663 possible responses, 248 (37.4%) quizzes were completed, again with the highest percentage of weeks completed by PGY3 residents (42.6%). The average individual weekly time engagement was 5.50 minutes (range 2.3–20.0). Out of a self-reported weekly studying time of 71.6 minutes, the study tool made up 7.7% of resident’s study time per week for those who used it. Our timestamp measure of engagement is crude as it only measures time with the form open; however, the observed time was consistent with the expected engagement, indicating reasonable accuracy.

Eighteen of 39 residents (46.0%) completed the post-intervention survey, with 15 of them having participated at least four times, and one having completed no repetition weeks. Given the low overall participation, we examined reasons for not participating. Respondents reported difficulty finding time to complete the quiz (33%) and missing the weekly email (28%) as the most common reasons. The tool was reported as helpful to both reinforcing academic half-day content (7.94/10, range 5–10) and to learning in general (7.89/10, range 5–10), though we were missing perspectives from most low frequency tool users.

Despite low participation, we still wanted to assess the effect of our intervention on learning. The total number of complete reinforcement cycles (tool usage on days 1, 8, and 29 following a given half-day lecture) was 93. This was distributed across 23 participants (range 1–11; m = 3.6). The average score on reinforced questions was 63.4% (95% CI 53.6–73.3), and on unreinforced questions 65.5% (95% CI 53.7–73.2), with no statistically significant difference found between the two groups. We observed a favourable trend towards improved scores on reinforced question content from PGY1 to PGY3 (see Fig. 1 of the Electronic Supplementary Material), though we were underpowered to find statistical significance.

## Lessons learned

Durable participation in the curriculum companion was limited, with only one third of residents using the tool for more than half of all opportunities. Participation across training year was relatively consistent, though we did not assess what made individual residents more or less likely to use the tool consistently. Reported reasons for missing opportunities seemed to primarily be related to the residents’ busy work schedules, as illustrated by their reports of missing emails, and lacking time to spend 5–10 minutes on high-yield, curated studying opportunities. Indeed, other authors have commented on the multiple draws on a resident’s time [14].

We did not incentivize participation, hoping to obtain a raw measure of engagement based on perceived learning value alone; we were surprised that in our population, durable participation due to intrinsic value of the intervention to learning was limited, as the value of spaced repetition had been reviewed with all residents on multiple occasions. Given the competing demands on a resident’s time, we hypothesize that improved participation could be achieved with more explicit programs of incentivization. To this end, we are conducting a follow-up study that applies gamification principles to the intervention to add elements of fun, teamwork, and competition. We hypothesize that this will improve both engagement in, and the efficacy of our intervention.

Also surprising was that we observed a trend in average score in favour of the unreinforced material, though this was not found to be statistically significant for pedagogically relevant differences. This may have been due to a number of reasons. First, a single MCQ may have been too specific a measure to reflect all learning achieved through the lecture, key points, and day 8 MCQ with disambiguation. Perhaps a more comprehensive assessment of content covered, such as a block exam, would better delineate differences on the basis of participation in the intervention. Second, any theoretical benefit may have been smaller than the desired improvement of one standard deviation. Scores appeared higher when fully reinforced (63.44%) than in the overall question set (60.93%) compared with the unreinforced question set, which were unchanged (65.59% versus 65.56%). Given the proven efficacy of spaced repetition [7–9], and test-enhanced learning [7, 15] we believe that limited participation likely confounded our results and dampened impact.

Our study was designed to have power to detect an 8.5% difference with 16 participants. We were therefore powered to detect a difference; however, only 13 residents participated in more than half of the weeks. This likely introduced self-selection and response bias for our secondary objective, as each individual represented a greater impact on the overall final result (for example, only five residents represented 54.8% of all analysed responses). Finally, it is also possible that two reinforcements are not enough to establish durable retention, as more questions are more beneficial to establishing test-enhanced learning [16]. A recent randomized controlled trial using electronically delivered MCQs to enhance learning in a paediatric emergency medicine rotation also found no difference between residents who had or had not received test-enhanced learning, in spite of high participation [17]. Though spacing of test questions was present in that trial, there was no spaced repetition, which may be another critical component when assessing distant recall. Our study used both strategies, but was impaired by poor participation, while their study had better participation but used only test-enhanced learning.

Interestingly, while performance on unreinforced questions is steady across years, PGY3 residents seemed to derive more benefit from reinforcement (not statistically significant) than did PGY1 and PGY2 participants. This may reflect increased clinical exposures, allowing them to experience our reinforcement tool as additional repetitions on top of previous ones. By comparison, PGY1 and PGY2 participants have simply not had enough exposures to create the foundation that effective spaced repetition is built upon.

Finally, participants reported a high level of satisfaction with the study tool, with all respondents expressing a desire to see it continue as an adjunct to the academic half-day. These results are mostly from those who used the tool regularly and are therefore subject again to self-selection bias. This intervention was unsuccessful in reinforcing or increasing learning, as measured, for the majority of residents. It remains important to note that this is in spite of high learner satisfaction, highlighting the fact that satisfaction alone is not an adequate marker of success in pedagogical intervention.

## Moral of the story

Our academic half-day study intervention was perceived as helpful and required a limited amount of learner’s time but was only used consistently by just over one third of all residents. Despite its basis in well-established pedagogy the effectiveness of our curriculum companion in its current form is unproven. We were surprised to identify the significant disconnect between high learner satisfaction with the intervention, and the disappointing level of learner participation and subsequent impact. To this end, educational quality improvement and research should not rely on learner satisfaction alone as a marker of success. Finally, failure to recognize and plan for operational barriers such as the overwhelming draws on a resident’s time and attention are likely to be the downfall to achieving satisfactory participation in many projects.

## Supplementary Information

Fig. 1 Scores on multiple choice questions with complete reinforcement cycles versus unreinforced questions, along with subgroups by post-graduate year. PGY, Post-Graduate Year.
