# Supplementary material for: Engagement and learning in an electronic spaced repetition curriculum companion for a paediatrics academic half-day curriculum
Source: Perspect Med Educ. 2021 Sep 13;10(6):369–72. doi: 10.1007/s40037-021-00680-x (PMC8633189; doi:10.1007/s40037-021-00680-x)
Supplement: Supplementary file 1 — Fig. 1 Scores on multiple choice questions with complete reinforcement cycles versus unreinforced questions, along with subgroups by post-graduate year. PGY, Post-Graduate Year. [file 40037_2021_680_MOESM1_ESM.docx]

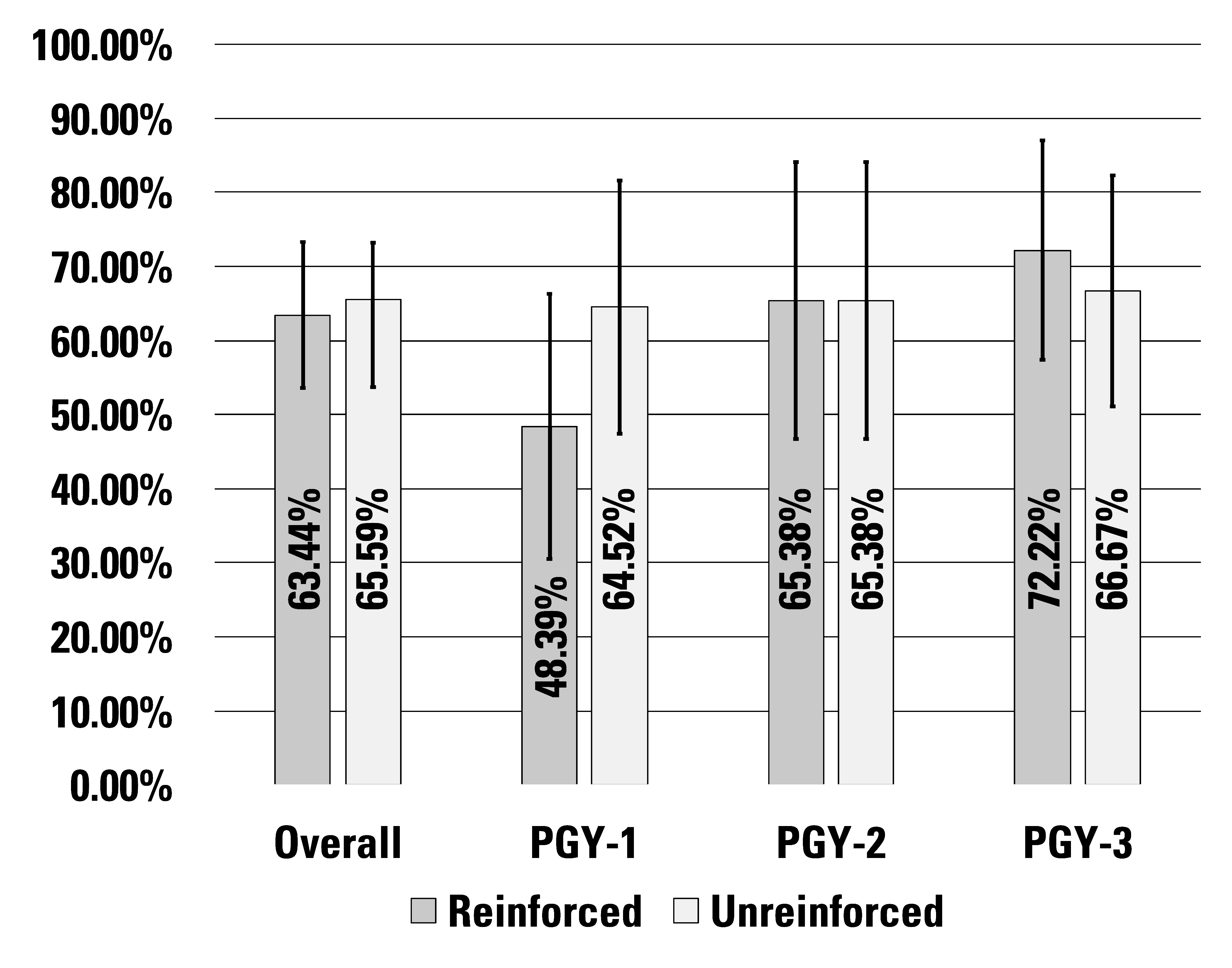


**Figure 1.** Scores on multiple choice questions with complete reinforcement cycles versus unreinforced questions, along with subgroups by post-graduate year. PGY, Post-Graduate Year.
